# Development and validation of a novel Spectrofluorimetric method of oral anticoagulant Edoxaban via derivatization with 9-fluorenyl methyl chloroformate: green assessment of the method by Eco-Scale and ComplexGAPI

**DOI:** 10.1186/s13065-022-00890-2

**Published:** 2022-11-23

**Authors:** Mohamed Rizk, Maha Mahmoud Abou El-Alamin, Ola Abd Elkhalek, Aliaa I. Shallan

**Affiliations:** Department of Pharmaceutical Analytical Chemistry, Faculty of Pharmacy, Helwan University, P. O. Box 11795, Cairo, Egypt

**Keywords:** Anticoagulant, Edoxaban, Spectrofluorimetry, Eco-Friendly, FMOC-Cl

## Abstract

A precise, sensitive eco-friendly, simple, rapid, and derivative spectrofluorimetric method was developed to quantify edoxaban tosylate monohydrate in pure form and pharmaceutical dosage form. Sudden death due to pulmonary embolism as a consequence of coronavirus infection (covid-19) is an emerging problem. As a result, the world health organization introduced new guidelines to treat patients with COVID-19 with oral anticoagulants. Edoxaban tosylate monohydrate is an oral anticoagulant that doesn’t require hospitalization after dose adjustment. This spectrofluorimetric method relies on the derivatization by 9-fluorenyl methyl chloroformate at room temperature in borate buffer pH 9.0. After excitation at 265 nm, the product is highly fluorescent at 309 nm. Many experimental factors influencing the reaction's stability and development were thoroughly investigated and optimized. The method validation was evaluated by using ICH guidelines and showed high precision and accuracy with an average percent recovery of 101.46% ± 1.02. The linear range was 5.0–50.0 ng/mL with a correlation coefficient of 0.9999, the LOD was 1.5 ng/mL, and the LOQ was 4.5 ng/mL. The green assessment of the method was achieved utilizing the eco-scale and the Green Analytical Procedure Index. There was no significant difference between the results of the suggested method and those of the reported method according to Statistical analysis.

## Introduction

Edoxaban tosylate monohydrate (EDTM) is one of the Novel Oral Anti-Coagulants (NOACs) class of drugs that are used in the treatment of pulmonary embolism (PE) as a result of COVID-19. The coronavirus disease (COVID-19) is because of infection with severe acute respiratory syndrome (SARS-CoV-2) [1], many reports have described coagulation events, such as fetal PE in these patients. EDTM was an effective and safe treatment for this acute (PE) in COVID-19. It is a selective factor Xa inhibitor which can be utilized to lower the risk of stroke caused by a blood thrombus in people who have atrial fibrillation. It is also utilized to treat deep vein thrombosis.

**Fig. 1:**
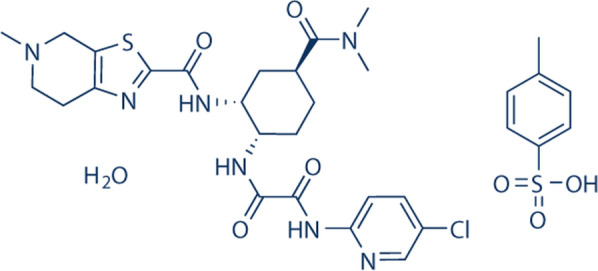
The molecular structure of EDTM

The IUPAC chemical name for EDTM is *N*′-(5 chloropyridin-2-yl)-*N*- [(1*S*,2*R*,4*S*)-4-(dimethyl carbamoyl)-2-[(5-methyl-6,7-dihydro-4*H*-[1,3]thiazole[5,4-c]pyridine-2-carbonyl)amino]cyclohexyl]oxamide; 4-methyl benzene sulfonic acid. The molecular structure of EDTM is presented in Fig. 1. Several methods were reported as UV Spectrophotometric [2, 3], HPLC [4–9], UHPLC-MS/MS [10–12], LC–MS/MS [13], HPTLC [14] and potentiometric method by a glassy carbon electrode [15]. The previously published works revealed that there was no reported spectrofluorimetric method for EDTM quantification. The spectrofluorimetric method remains the most convenient analytical technique due to its low cost, simplicity, and widespread availability in most quality control laboratories in the developing countries instead of chromatographic methods which require expensive and sophisticated solvents and instruments that may be not available in some of quality control laboratories. EDTM is not fluorescent and only a fluorescent derivative reaction could be used to determine its fluorescence. 9-Fluorenylmethyl chloroformate chloride (FMOC-Cl) is an important derivatizing agent utilized to determine primary, secondary amines [16] and many pharmaceutical compounds [17–25]. EDTM has secondary amine groups, so we select FMOC-Cl as a fluorogenic reagent to derivatize EDTM. In this paper, we aimed to develop a sensitive, simple, accurate, rapid, and derivative spectrofluorimetric method for EDTM quantification in tablet. This method is distinguished by its low chemical and energy consumption, making it one of the promising green analytical techniques.

## Experimental

### Instrumentation

JASCO FP-6200-Spectrofluorimeter using 1.0 cm quartz cells, a 150 W xenon lamp and, Instrument emission and excitation slits were both optimized to 10 nm. Spectra were assessed by FP-6200 control Driver software, Version1.54.03 (Build 1), JASCO Corporation. A digital pH meter (Hanna HI-2211) was used for pH adjustment and bench-top sonicator; USA.

### Reagents and materials

EDTM pure sample was kindly obtained from Rameda pharmaceutical company (Cairo, Egypt). Coaguloban^®^ (Marcyrl Company) 60 mg was purchased from the local market, 9-fluorenylmethyl chloroformate (FMOC-Cl), (St. Louis, USA), a freshly prepared stock solution containing 10 mg% w/v of FMOC-Cl in acetonitrile was diluted with acetonitrile also to produce 0.01 mg% w/v solution, monobasic potassium phosphate, sodium hydroxide, boric acid, potassium chloride, sodium acetate and glacial acetic acid were obtained from El-Nasr Pharmaceutical Chemicals Company (Cairo, Egypt). According to USP Pharmacopeia, 0.02 M acetate buffer was prepared to cover pH range from 4.0 to 6.0, 0.02 M borate buffer covering pH range from 8.0 to 10.0, and 0.02 M phosphate buffer pH range from 6.0 to 9.0 solutions was prepared by adding 50 mL of 0.2 M monobasic potassium phosphate in 200-mL volumetric flask then added appropriate volume of sodium hydroxide solution and diluted to 100.0 mL with water. Acetonitrile and methanol were of HPLC grade (99.9%). Double distilled water was utilized throughout the procedure.

### Standard solution

#### Stock solution

100 mg of EDTM was weighed and transferred into 100 mL volumetric flask, then the volume was adjusted by methanol for the preparation of 1.0 mg/mL of EDTM solution and it is stable for 3 days when kept at 4.0 °C.

#### Working solution

50.0 µL of stock solution was transferred into 50 mL volumetric flask, then the volume was completed by double distilled water to prepare a solution of 1.0 μg/mL of EDTM.

### Procedures

#### Construction of calibration graph

Aliquots of EDTM working solution equivalent to 5–50 ng/mL were added into a set of 10 mL volumetric flasks. Following the addition of 2 mL of borate buffer pH 9.0, 0.5 mL of 0.01% FMOC-Cl was added, thoroughly mixed and 2 mL of Acetonitrile was added also. After 15 min at room temperature, the reaction mixture was completed to the mark with distilled water. After excitation at 265 nm the fluorescence emission of the generated derivative was measured at 309 nm. A blank experiment was done similarly. To get the calibration graph the corrected fluorescence intensity was plotted against the final drug concentration (ng/mL).

#### Application of the suggested method for analysis of EDTM in dosage forms

Ten tablets of coaguloban^®^ were weighed, finely powdered, mixed well and an accurately weighed amount of the powder equivalent to about 15 mg EDTM was accurately added to a conical flask and dissolved in 50 mL methanol, The flask was sonicated for 30 min, the solution was filtered and transferred quantitatively to 100 mL volumetric flask and the volume completed with the distilled water. Aliquots covering the range of (50–500 ng/mL) were added to a set of 10 mL volumetric flasks, and the method for the calibration graph mentioned above was then carried out.

## Results and discussion

In this work we provided a novel, simple, precise, rapid, sensitive, and low-cost spectrofluorimetric method for the determination of EDTM in pure form and tablets. we optimized the derivatization parameters to improve the yield of the derivatization reaction.

**Fig. 2:**
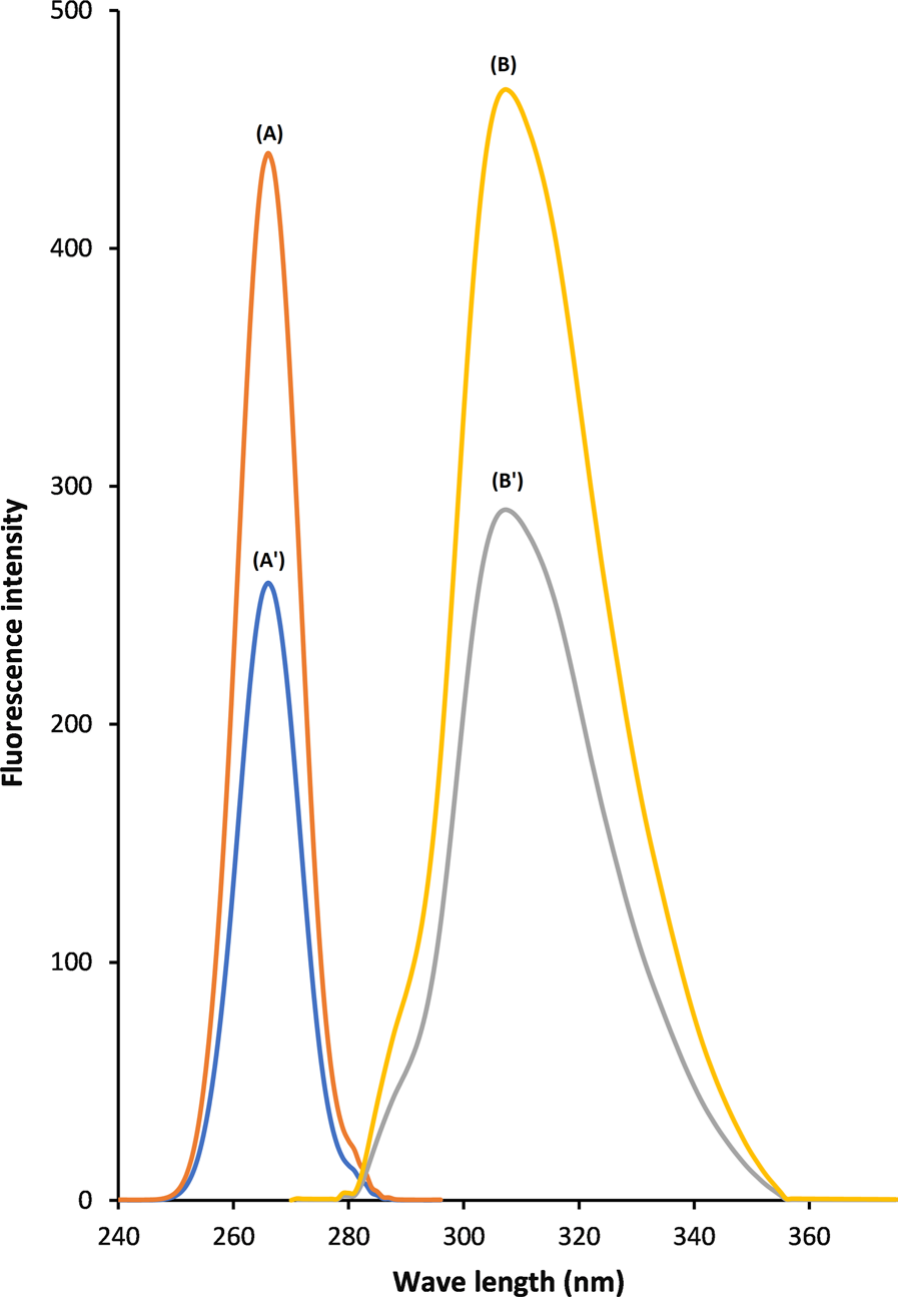
Fluorescence spectra of emission and excitation of blank and EDTM-FMOC, (*A*) and (*B*) Excitation and emission spectrum of EDTM (15 ng) with FMOC-Cl at pH 9.0. (*A*′) and (*B*′) Excitation and emission spectrum of the blank

FMOC-Cl was selected as a fluorogenic derivatizing agent for EDTM due to its preferable characteristics and stability of the yield of derivatization. Its reaction occurs in an alkaline medium under mild conditions which makes it superior to other derivatizing agents. It has been used to determine pharmaceutical compounds containing primary and secondary amines [16, 26, 27]. In this work, EDTM was observed to react with FMOC-Cl in borate buffer pH 9.0 forming a highly fluorescent derivative with λ maximum at 309 nm after excitation at 265 nm as shown in Fig. 2.

**Fig. 3:**
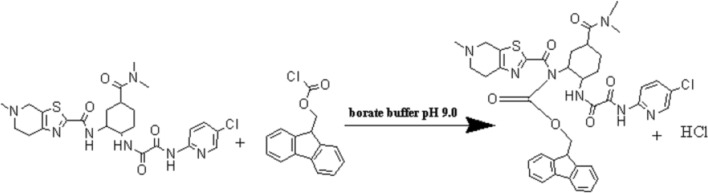
The suggested derivatization reaction between EDTM and FOMC-Cl

This occurs by the substitution of a hydrogen atom in the secondary amine group in the EDTM by the aromatic rings of FMOC-Cl resulting in the formation of FMOC-EDTM. The suggested derivatization reaction between EDTM and FMOC-Cl is shown in Fig. 3.

### Optimization of the experimental conditions

The spectrofluorimetric characteristics of the generated derivative, the various experimental factors influencing the derivatization reaction of EDTM and its stability were carefully investigated and optimized. These variables were altered separately while the others remained constant. The experimental parameters included pH, type of buffer, FOMC reagent volume, buffer volume, acetonitrile volume, and reaction time. Each factor was measured three times.

#### The influence of pH

**Fig. 4:**
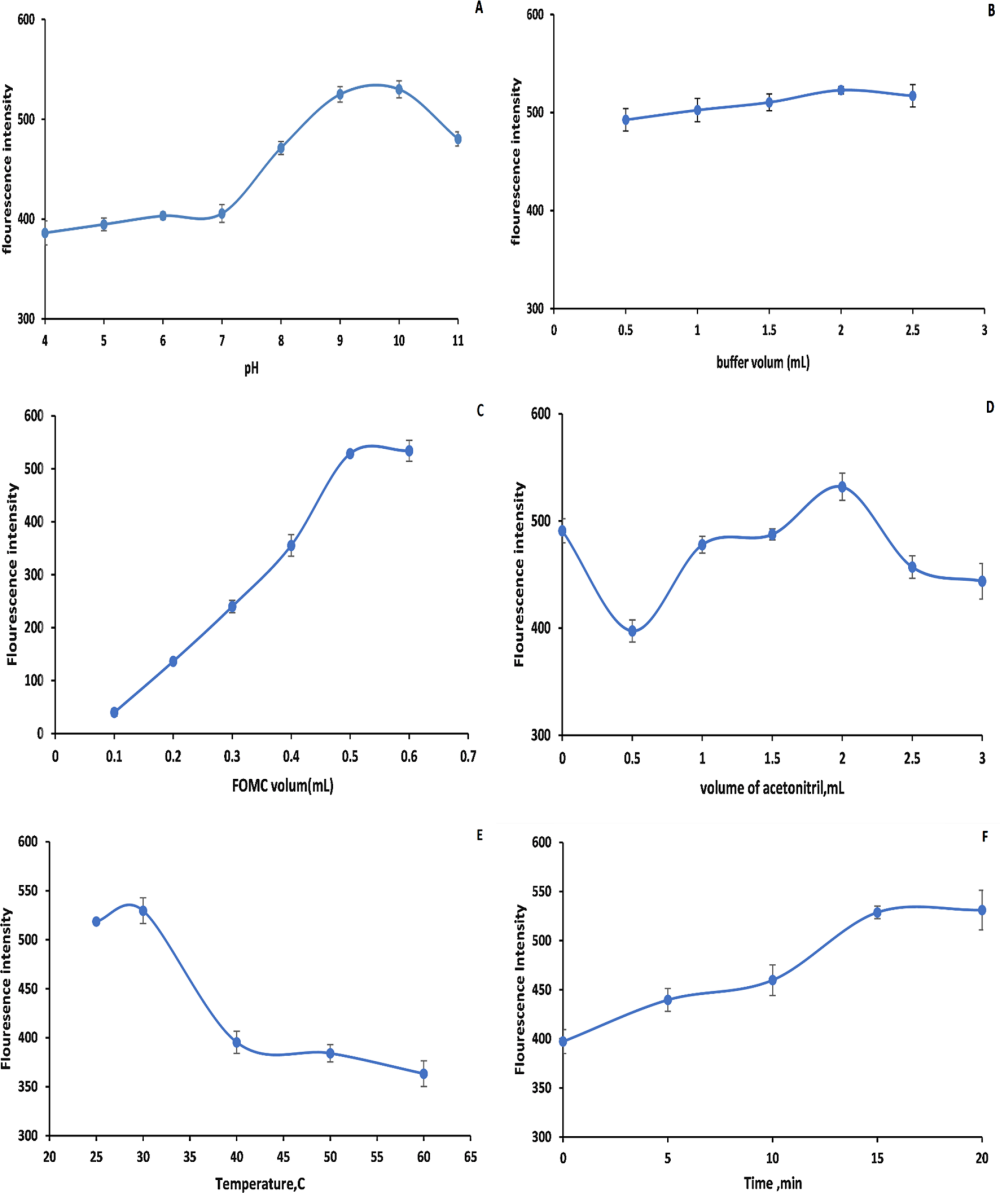
Optimization of experimental conditions for EDTM reaction with FMOC-Cl each factor was measured three times including: **a** The influence of buffer pH on the formation of EDTM-FMOC after derivatizing of EDTM (15 ng/mL) with FMOC-Cl. **b** The influence of buffer volume on the formation of EDTM-FMOC between EDTM (15 ng/mL) and FMOC-Cl. **c** The influence of FMOC-Cl volume on the formation of EDTM-FMOC between EDTM (15 ng/mL) and FMOC-Cl. **d** The influence of volume of acetonitrile on the formation of EDTM-FMOC between EDTM (15 ng/mL) and FMOC-Cl. **e** The influence of heating temperature on the formation of EDTM-FMOC between EDTM (15 ng/mL) and FMOC-Cl. **f** The influence t of heating time on the formation of EDTM-FMOC between EDTM (15 ng/mL) and FMOC-Cl

The influence of pH was firstly investigated due to its significant impact on the fluorescence intensity of the generated derivative. Several basic pH was tried to determine which pH has the maximum fluorescence intensity of EDTM-FMOC derivative. pH 9.0 maximum fluorescence intensity was obtained and remained constant up to 10.0 after which the fluorescence intensity of the reaction product started to reduce gradually until pH 11.0. So, pH 9.0 was selected as the ideal pH as shown in Fig. 4a another buffer that has the same pH value (9.0) was tried as 0.2 M phosphate buffer and compared with 0.2 M borate buffer. Borate buffer was observed to be more effective than phosphate buffer. For the maximum intensity of the reaction product, 0.2 M borate buffer at pH 9.0 was optimal.

#### The influence of buffer volume

The intensity of the generated derivative is significantly affected by the buffer volume. It was observed that increasing the volume of 0.2 M borate buffer of pH 9.0 up to 2 mL led to a gradual improvement in the fluorescence intensity of the generated derivative so 2 mL of the buffer was selected as the ideal volume during the procedure as it is shown in Fig. 4b.

#### The influence of FMOC-Cl volume

The effect of FMOC-Cl volume was investigated utilizing many volumes of 0.01 mg% of the reagent solution. The derivatization reaction of EDTM with FMOC-Cl began when 0.1 mL of FOMC-Cl reagent was added in the presence of borate buffer (pH 9.0). The fluorescence intensity of the EDTM-FMOC derivative increases proportionally to the reagent volume up to 0.5 mL and maintains its consistency up to 0.6 mL. As a result, 0.5 mL of 0.01 mg% of FMOC-Cl solution was selected as the ideal volume of the reagent as presented in Fig. 4c.

#### The influence of acetonitrile volume

2 mL of acetonitrile was found to be adequate to produce the highest and most consistent fluorescence intensity of the EDTM-FMOC derivative. Because of the insolubility of FMOC-Cl and the generated derivative in an aqueous medium, the derivatization reaction between FMOC-Cl and EDTM occurs in an aqueous-organic medium. The reaction had to be carried out with a high content of acetonitrile as presented in Fig. 4d.

#### The influence of temperature

The influence of temperature on the formation of the reaction product was studied by heating the reaction mixture over the temperature range (25–60) increasing the reaction temperature above 30 °C led to a reduction in the fluorescence intensity of EDTM-FMOC as presented in Fig. 4e.

#### The influence of reaction time

Several time intervals were investigated to know when the solution reached its peak fluorescence. After 15 min, the EDTM-FMOC derivative had the maximum fluorescence intensity and stayed stable up to 20 min (Fig. 4F). As a result, the generated derivative was measured after 15 min at 30 °C temperature.

### Validation of the suggested method

**Fig. 5:**
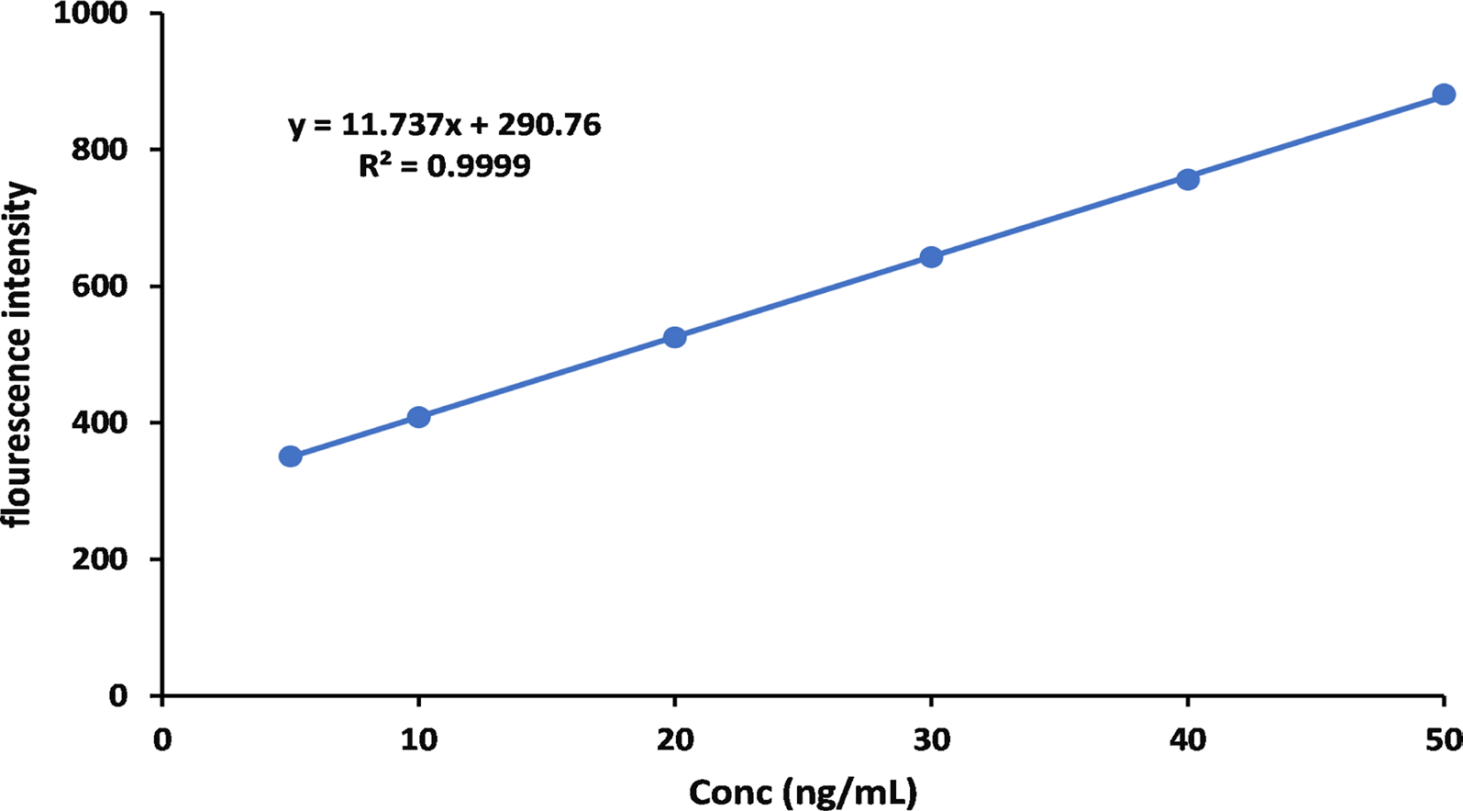
Calibration curve

The suggested method was validated according to ICH guidelines [28] concerning linearity, range, LOD, LOQ, selectivity, precision, and accuracy. The calibration curve is shown in Fig. 5.

#### Linearity and range

A linear relationship was obtained between the concentration of the studied drug over the concentration ranging from 5 to 50 ng/mL and fluorescence intensity. The regression equation and parameters were calculated and listed in Table 1. a linear regression analysis of the data gave the following equation:

$$ F = 11.737C + 290.76 \, \left( {R^2 = \, 0.9999} \right) $$

*C* = the concentration of the studied drug in ng/mL; *F* = the fluorescence intensity; *R*^2^ = coefficient of determination.

**Table 1:** Performance data of the suggested method to determine EDTM in pure form

Concentrations (ng/mL)	Fluorescence intensity
5	350.761
10	408.153
20	525.016
30	642.495
40	756.297
50	880.997

Parameter	Value
Regression equation	*Y* = 11.737*X* + 290.76
Linear range (ng/mL)	5–50 ng/mL
Coefficient of determination (R^2^)	0.9999
%Recovery	101.46
Relative standard deviation (%RSD)	1.003
Standard deviation (± SD)	1.02
Limit of quantitation (LOQ)	4.5
Limit of detection (LOD)	1.5
Percentage error (%Er)	0.004

#### Limit of quantification (LOQ) and detection (LOD)

Limit of quantification (LOQ) and detection (LOD) were calculated as shown in Table 1. The limit of quantification is the lowest concentration that can be determined according to ICH Q2(R1) [29] according to this equation (LOQ = 10*σ/S*) where σ is the standard deviation of the intercept of the regression line of the calibration curve and *S* is the slope of the linearity. The limit of detection (LOD) is the lowest concentration of the analyte that can be detected and calculated in accordance with this equation (LOD = 3.3*σ/S*).

#### Accuracy

The accuracy of the suggested method was studied by determining percent recoveries and percentage error as shown in Table 1. The results represent good recoveries and small percent relative error that indicate good accuracy of the suggested method.

#### Precision

The precision of the suggested method was investigated by using three different concentrations of the standard solution of EDTM each concentration was measured in triplicate on three successive days to determine interday precision (intermediate precision), while intraday precision (repeatability) determined by measuring each concentration of standard solution of EDTM (15, 30 and 45 ng/mL) in triplicate within the day. The results presented in Table 2 show low values of SD, %RSD, %E which indicate high precision of the suggested method.

**Table 2:** Accuracy and precision data of the suggested method to determine EDTM in pure form

Parameter	Intra-day precision (repeatability)			Inter-day precision (intermediate precision)		
Concentration (ng/mL)	15	30	45	15	30	45
%Recovery	99.62	99.07	100.55	99.77	99.19	100.69
± SD	1.02	1.34	1.29	1.34	1.22	1.53
%RSD	1.022	1.36	1.29	1.35	1.23	1.52
%E	0.59	0.78	0.74	0.77	0.71	0.88

### Pharmaceutical application

The applicability of the suggested method was evaluated by determining EDTM in tablet dosage form. The results in Table 3 represented that the average recovery was 99.78 ± 1.18% reflecting the high precision and accuracy of the suggested method. The results achieved were statistically compared with a reference method using Student’s *t* test and variance ratio *F*-test [30] as presented in Table 4. The results show that there is no significant difference between the two methods.

**Table 3:** Precision and accuracy of the suggested method to determine EDTM in the tablet

Parameter	Intra-day precision			Inter-day precision		
Concentration (ng/mL)	15	30	45	15	30	45
%Recovery (Coaguloban^®^ tablets 60 mg/L)	99.62	99.29	100.84	99.48	98.55	100.91
±SD	1.29	1.34	1.84	1.25	1.13	1.15
%RSD	1.30	1.35	1.82	1.26	1.15	1.15
%*E*	0.75	0.78	1.05	0.73	0.66	0.66

Each result is the average of three measurements

**Table 4:** Statistical comparison between suggested method and reference method to determine EDTM in the pharmaceutical dosage form

Parameter	Suggested method	Reference method [8]
	99.48	99.81
	98.55	99.76
	100.92	99.36
	99.62	
	100.84	
Mean (%) ± SD	99.88 ± 0.99	99.64 ± 0.25
*t*-test	0.39 (2.45)	
*F*-test	16.55 (19.25)	

The value in brackets is the theoretical ones at *P* = 0.05

## Reference method using high-performance liquid chromatography (HPLC)

The laboratory protocol for the reference method requires the validation of the reference method before using it in the analysis of the drug. Hypersil BDS C18 column was used with an eluting solvent consisting of 0.1 M K_2_HPO_4_: Methanol (65:35, v/v) as an isocratic mobile phase at a flow rate of 1.0 ml/min. The calibration curve was obtained by plotting peak area (*A*) against concentrations (*C*). the obtained linear range was *A* = 19,702*C* + 24,446 with regression coefficient (*R*^2^) = 0.9998. The absorbance of the eluted drug was measured by a photodiode array set at 245 nm. The calibration curve was linear over the concentration range 5–200 μg/mL. The accuracy of the method ranged from 99.824 to 100.72% and the precision was ≤ 0.71%.

## Comparative analysis

By comparing our suggested method with the recently reported methods as presented in Table 5. The results represented that our method is sensitive, rapid, and instrumentally affordable compared to the other methods such as the chromatographic method, which is an expensive method, requires a complicated pretreatment process, requires a large number of expensive organic solvents, and is not available in all quality control laboratories, especially in developing countries.

**Table 5:** Comparison between the suggested method and the recently reported methods

Method	Linear range	LOD	References
Spectrophotometric	5–25 µg/mL	0.654 μg/mL	[2]
HPLC–UV	2–10 µg/mL	0.225 μg/mL	[5]
Potentiometric	3.288–548.056 µg/mL	1.858 μg/mL	[15]
Our suggested method	5–25 ng/mL	1.49 ng/mL	

## Green assessment of the method

Analytical methods use a lot of chemicals that may generate toxic residues to the environment. Herein the green analytical chemistry was first presented in 2000 to remove or reduce the hazardous effect on the environment [31]. various assessment tools were introduced to evaluate the greenness of analytical method depended on wastes, energy consumption, and solvents used per study. Each tool has a unique assessment protocol advantages and disadvantages so in this study we applied two assessment tools in green assessment as the analytical eco-scale assessment (ESA) [32] and the newest tool in green assessment called complex green analytical procedure index (ComplexGAPI).

ESA has the advantage of providing a quantitative evaluation of the analytical methods by considering all of the reagents used, rather than just the most hazardous ones, as other matrices do. It based on penalty points subtracted from a 100 mark a total eco scale score if more than 75 represents excellent greenness, more than 50 represents acceptable greenness and less than 50 represents inadequate greenness Table 6 represents the scores for the suggested method. Our method achieved 87 penalty points, which demonstrated its excellent greenness. It has several disadvantages including insufficient information about the causes of the analytical procedure’s environmental impact and no data about the structure of the hazards is founded. Our method is regarded as an excellent green analysis.

**Table 6:** Green assessment of our proposed method by Eco-Scale and Complex-GAPI

	Eco-scale parameter	Penalty points	Diagram of complex gapi
Reagents	FMOC-Cl	2	
	Acetonitrile	2	
	Boric acid	2	
	Sodium chloride	2	
	Methanol	1	
Instrument	Spectrofluorometry	0	
	pH meter	0	
	Sonicator	1	
	Occupational hazard waste:	0	
	10–100 mL	3	
	Degradation	0	
∑penalty		13	
Total score		87 Excellent greenness	

ComplexGAPI is the most recent advanced technique or assessment of green chemistry. It is the developed version of GAPI assessment. It represents a comprehension evaluation of the whole analytical process from sample collection to final analysis including transport, preservation, storage, and sample preparation. The scale is based on utilizing five pentagrams and the additional hexagonal part is the pre-analysis step. Green color represents an eco-friendly step yellow color represents a medium environmental impact and red color represents a hazardous environmental impact of this step [33]. There is no need for a purification step, which corresponds to the white region in the hexagonal shape. This technique has a lot of advantages as the software is available that will facilitate the use of such a tool, it is simple, friendly use, and includes all the factors that characterize the analytical protocol as well as the pre-analysis process (conditions, techniques, and reagents). Our method is eco-friendly according to the application of two approaches eco-scale and complexgapi as presented in Table 6.

## Conclusion

The suggested method is accurate, simple, precise, sensitive, eco-friendly, rapid, and appropriate for routine testing in quality control departments. It is appropriate to determine EDTM in pure form and tablet. Due to the stability and simplicity of the derivatization reaction, it can be used for the pretreatment of EDTM drugs for chromatographic analysis in biological fluids with high sensitivity. This method is also eco-friendly and suitable for use in quality control departments with the elimination of carcinogenic and hazardous chemicals.

## Data Availability

The data that support the findings of this study are available from the corresponding author upon reasonable request.
